# Ecological Momentary Assessment of Fatigue in Adults with Cerebral Palsy: Feasibility, Reliability, and Validity

**DOI:** 10.3390/brainsci16050515

**Published:** 2026-05-12

**Authors:** Frederik Have Dornonville de la Cour, Sun-Hee Skovgaard Christensen, Stine Flensburg Hansen, Anne Norup

**Affiliations:** 1 Neurorehabilitation Research and Knowledge Centre, Copenhagen University Hospital–Rigshospitalet, 2600 Glostrup, Denmark; fdor0002@regionh.dk (F.H.D.d.l.C.); sun-hee.skovgaard.christensen@regionh.dk (S.-H.S.C.); 2 Department of Neuroscience, University of Copenhagen, 2200 Copenhagen, Denmark; 3 Elsass Foundation, 2920 Charlottenlund, Denmark

**Keywords:** real-time fatigue, neurological disorders, neurorehabilitation, experience sampling method, real-time monitoring, intensive longitudinal methods, ambulatory assessment, psychometric properties, person-specific reliability, compliance

## Abstract

**Highlights:**

**What are the main findings?**

- High-frequency ecological momentary assessment of fatigue is feasible in adults with cerebral palsy, with no evidence of reactivity to self-monitoring;
- More than half of the total variability in fatigue occurred within persons, and nearly three quarters of these fluctuations were systematic rather than measurement error.

**What are the implications of the main findings?**

- Intensive real-time monitoring is justified in this population, as retrospective assessments cannot capture the temporal dynamics of fatigue in everyday life;
- These findings inform a future large-scale study in cerebral palsy, with implications for understanding symptom mechanisms and advancing fatigue management.

**Abstract:**

**Background/Objectives**: Fatigue is a common symptom in adults with cerebral palsy (CP), characterized by fluctuations across the day. This pilot study aimed to evaluate the feasibility, reliability, and validity of ecological momentary assessment (EMA) for capturing these temporal dynamics in adults with CP. **Methods**: Ten adults with CP (60% female, mean age = 44 years, Gross Motor Function Classification System levels I–III) and eight typically developed controls (62% female, mean age = 39 years) completed a 20-item EMA survey ten times daily for seven days using the SEMA^3^ smartphone application. Feasibility was evaluated through retention rates, response rates, and qualitative interviews. Intraindividual variability, within-person reliability, measurement reactivity, and convergent validity with the Fatigue Severity Scale (FSS) were examined using mixed-effects regression and multilevel measurement error autoregressive (MEAR) models. **Results**: No participants dropped out. Average response rates were 76% (CP) and 75% (control). The protocol was perceived as acceptable overall, though demanding by some participants. In the CP group, 61% of total variability in momentary fatigue was attributable to within-person fluctuations, and within-person reliability was 0.73 (SEM = 1.13). No evidence of reactivity to self-monitoring was found in fatigue ratings or qualitative interviews. FSS scores were positively associated with person-level average momentary fatigue, β = 0.51, *p* = 0.048. **Conclusions**: EMA is feasible in adults with CP and reveals substantial within-person fluctuations in fatigue. These findings provide initial proof-of-concept and inform methodological amendments for a future large-scale study of fatigue dynamics aiming to advance symptom management in this population.

## 1. Introduction

Cerebral palsy (CP) is an “early-onset lifelong neurodevelopmental condition characterized by limitations in activity due to impaired development of movement and posture” [1] (p. 704). Fatigue is a particularly common and burdensome impairment for adults with CP [2], affecting about half of the population and contributing substantially to activity limitations and participation restrictions [3,4]. Importantly, fatigue has been identified as a key priority for research and clinical care by persons with CP, caregivers, professionals, and advocates [2]. It is defined as “a subjective lack of physical and/or mental energy that is perceived by the individual or caregiver to interfere with usual and desired activities” [5] (p. 2). Fatigue can present physically, cognitively, or emotionally, including muscular weakness or soreness, pain, poor concentration, or irritability.

Adults with CP describe that fatigue is dynamic and context-dependent, with severity fluctuating across days and within the day [3]. Unlike ordinary feelings of tiredness, fatigue may worsen dramatically and unexpectedly from one moment to the next, adding substantially to the overall burden of fatigue [3]. Several everyday factors are reported to exacerbate symptoms, such as physically or mentally demanding activities over extended periods of time, prolonged inactivity and sedentary behavior, noisy environments, and a high sensory load [6]. Yet, little is known about the dynamic nature of fatigue, and traditional questionnaires are inadequate to capture these patterns.

Ecological momentary assessment (EMA) is a set of methods for capturing everyday experiences in their immediate context, including thoughts, behaviors, and symptoms [7]. Respondents complete multiple assessments a day, as they go about their everyday lives, reporting on their symptoms and behaviors at the time. By sampling everyday experiences in real-time, EMA reduces bias and error attributable to memory limitations, personal beliefs, and self-concepts, and the longitudinal nature of EMA enables the assessment of dynamic processes and interactions occurring over short periods of time. Such fine-grained data are crucial for understanding how and why symptoms change during the day and for identifying the factors that may worsen or mitigate fatigue. This information may be used in clinical practice, providing the basis for a better understanding, treatment, and self-management of fatigue, through personalized feedback to patients about individual patterns of fatigue fluctuations and their triggers [8]. One study in acquired brain injury indicated that the process of self-monitoring and receiving visual feedback on the data could increase awareness of fatigue and factors that trigger or exacerbate fatigue and support the use of self-management strategies such as pacing [9].

However, a high sampling frequency is necessary to capture how fatigue unfolds across the day, which can be demanding for participants, and compliance may be particularly challenging for individuals with neurological conditions where fatigue, motor impairments, or cognitive demands may add to the burden of participation. Despite its potential, EMA remains underutilized in neurorehabilitation, and the reliability and validity of EMA-based measures have yet to be established for fatigue in this context [10]. The aim of this study was to explore the feasibility and measurement properties of EMA as a preparatory step for a large-scale study (ClinicalTrials.gov Identifier: NCT07135791). Specifically, we examined feasibility in terms of retention and compliance, as well as acceptability and perceived burden. To evaluate fatigue fluctuations and justify high-frequency EMA, we quantified the extent of intraindividual variability in momentary fatigue ratings over time, and we assessed the within-person reliability. We evaluated the risk of measurement reactivity by testing whether fatigue ratings change systematically across the monitoring period. Finally, we assessed the convergence between person-level aggregates of EMA fatigue ratings and a retrospective measure of fatigue to examine construct validity.

## 2. Materials and Methods

### 2.1. Study Design and Participants

This study employed an intensive longitudinal observational design followed by semi-structured follow-up interviews. The sample included adults with CP and adults without neurological conditions. Participants were recruited via the Elsass Foundation, a private Danish foundation supporting quality of life for people with CP. Study participation was advertised on the website and through word of mouth among individuals engaged in other activities at the foundation. Control participants were recruited from among Elsass Foundation employees and the researchers’ social networks.

Eligibility criteria for all participants were: (1) 30–65 years of age; (2) fluency in Danish; (3) ability to use a smartphone; and (4) no active drug or alcohol abuse. Additional inclusion criteria for participants with CP were: (1) self-reported CP diagnosis; (2) Gross Motor Function Classification System–Expanded and Revised (GMFCS) level I–III (able to walk with or without assistive devices) [11]; and (3) Communication Function Classification System (CFCS) level I–II (able to communicate independently with both unfamiliar and familiar partners) [12]. Control participants were required to have no neurological condition.

Informed consent and characteristics were collected remotely prior to real-time monitoring, using the REDCap electronic data capture platform 16.0.15 (Vanderbilt University, Nashville, TN, USA) [13,14].

### 2.2. Development of the Prototype EMA Items

**Figure 1:**
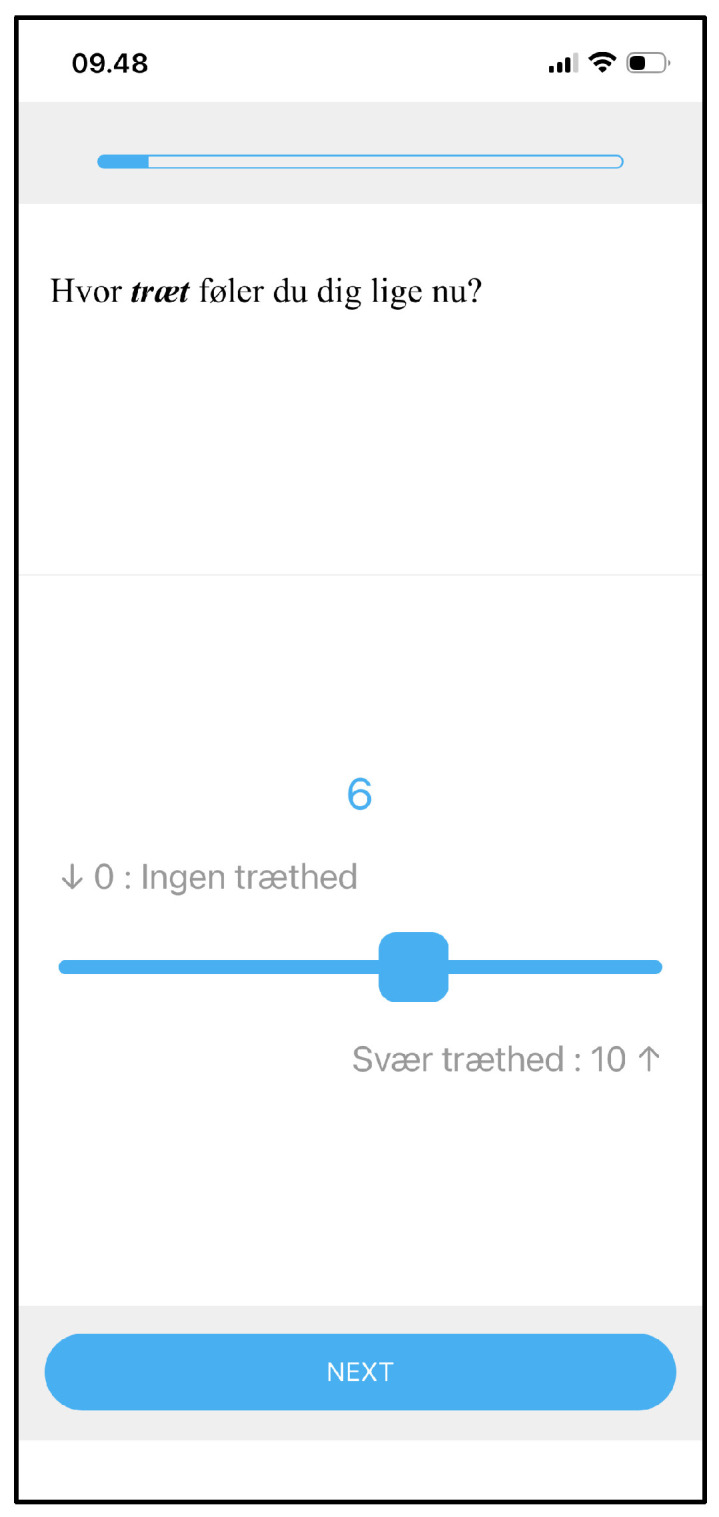
Screenshot of the momentary fatigue item in the SEMA^3^ app. In English, the question was “How ***fatigued*** do you feel right now?” with anchors defined as “↓ 0: No fatigue” (left-hand side) and “Severe fatigue: 10 ↑” (right-hand side).

A 20-item prototype EMA questionnaire was developed to assess the momentary, here-and-now, experience of fatigue, related factors, and contextual factors. The items were developed based on a literature review of EMA studies on fatigue, step-by-step guides to designing EMA items [15,16], and the ESM Item Repository [17]. Both positively and negatively worded questions (e.g., energy and fatigue) were used to avoid putting respondents in a negative mindset, only focusing on fatigue. A single-item indicator of momentary fatigue was defined as “how fatigued do you feel right now,” rated on a slider scale from 0 (no fatigue) to 10 (severe fatigue), as shown in Figure 1 (In Danish, the term “træthed” was used, which corresponds closely to “tiredness,” since there is no direct lexical equivalent of the English term “fatigue”). The intermediate response options were not labeled. This item has been used in previous studies in other populations, apart from small variations in anchors [18,19]. A follow-up question queried whether the experience of fatigue is primarily mental, physical, both, or neither. Additional items were selected to capture contextual information, related constructs, and potentially contributing, co-occurring, or confounding factors. These items included energy, exhaustion, pain, sleepiness, perceived effort required to do anything, concentration, and mood-related items, including happiness, relaxation, sadness, stress, and anxiety. Contextual items addressed the current activity, the motivation for engaging in that activity, the physical and mental demand associated with the activity, the current setting, sensory load, and social company (see Appendix A for a complete list of item wordings and response options).

### 2.3. The EMA Sampling Protocol

EMA data were collected using SEMA^3^ 1.4.0 (University of Melbourne, Melbourne, Australia; see Figure 1) [20]. Assessments were scheduled 10 times per day over 7 consecutive days, resulting in up to 70 measurements per participant. A time-stratified random sampling scheme was employed to mitigate potential reactivity effects while securing coverage from waking to bedtime. Assessments were triggered at random within 10 one-hour blocks, starting at 07:30 in the morning and ending at 22:00 in the evening. The scheme was adjusted by one hour if requested by the participant to accommodate personal bedtimes. When triggered, participants were prompted via a push notification on their smartphone to complete a survey in the SEMA^3^ app. The survey remained available for 30 min, with two reminders 15 and 2.5 min before expiry. Blocks were spaced 30 min apart to prevent overlaps, with intervals between successive prompts ranging from 30 to 150 min (mean = 90, *SD* = 25), depending on the randomization within blocks. The app displayed each participant’s current response rate on the home screen to encourage adherence. No monetary compensation or incentives were provided for participation or compliance.

On the day before data collection (Day 0), participants received a standardized briefing covering the rationale for real-time monitoring, the importance of maintaining usual daily routines, and instructions for carrying their smartphone with sound enabled throughout the week. They were asked to respond to notifications as soon as possible and to complete as many assessments as feasible. The SEMA^3^ app was installed, and a demonstration questionnaire was completed to ensure familiarity with app navigation, questions, and response formats. Situations in the coming week where responding might be difficult were addressed. A follow-up call on Day 2 was scheduled to address questions, mitigate any technical issues, and support adherence.

### 2.4. Retrospective Fatigue Questionnaire

The Fatigue Severity Scale (FSS) [21,22] was used as a retrospective measure of fatigue for validity analyses. The FSS is a 9-item self-report questionnaire about fatigue, e.g., “I am easily fatigued.” Each item is rated on a 7-point scale from 1 (strongly disagree) to 7 (strongly agree), and a mean score is computed with higher scores indicating greater fatigue severity. No time frame of reference was defined for the items. The FSS was administered prior to real-time monitoring.

### 2.5. Feasibility and Acceptability Outcomes

#### 2.5.1. Quantitative Feasibility Indicators

Adherence was assessed on the following metrics:

- Consent rate (% consenting to participate);
- Retention rate (% completing the study);
- Response rate (% of EMA surveys initiated);
- Completion rate (% of EMA surveys fully completed);
- Response latency (time from prompt to response initiation, based on time stamps);
- Completion time (time from response initiation to submission, in milliseconds).

To evaluate systematic trends of adherence, the proportion of responses was analyzed across (1) time-of-day, (2) study days, (3) weekend vs. weekday, and (4) time-lagged scores of momentary fatigue (*t*−1). Time-of-day was modeled according to the ten blocks of the standard EMA scheme, extended by one hour in each end to accommodate early and late schemes. Response latency was regressed on momentary fatigue ratings to examine whether higher fatigue was associated with longer delays between prompting and responding.

#### 2.5.2. Follow-Up Interviews

One-on-one interviews were conducted with participants with CP in the week after real-time monitoring to explore acceptability and perceived burden of the EMA protocol, including the relevance, clarity, and comprehensiveness of the items, with the aim of refining the protocol. We also asked participants to rate the extent to which real-time monitoring influenced their (1) mood, (2) daily routine, and (3) social interactions to evaluate perceived reactivity to self-monitoring.

A semi-structured interview guide was designed, including open-ended questions and a structured questionnaire. All interviews were audio-recorded and transcribed verbatim.

### 2.6. Statistical Analysis

EMA data were analyzed using multilevel models to account for the hierarchical structure with repeated assessments (Level 1) nested within days (Level 2) nested within persons (Level 3). Separate models were fit for each predictor, and fixed effects were tested by comparing nested models with and without the effect of interest using the likelihood ratio test, setting the significance level at α = 0.05.

#### 2.6.1. Intra-Individual Variability

To assess the dynamics of fatigue, the total variance of the EMA items was decomposed across levels using an empty model without predictors. The intraclass correlation coefficient (ICC) was calculated for both persons (Level 3) and days within persons (Level 2) to quantify the proportions of total variance attributable to differences between persons and to differences between days within persons, respectively [23]. The remaining variance is attributable to moment-to-moment fluctuations within the day (Level 1).

#### 2.6.2. Within-Person Reliability

Conventional reliability indices such as internal consistency and test-retest reliability are generally not suitable for EMA data, where constructs are often assessed with single items and the primary interest is within-person variation over time. Therefore, we estimated reliability using the multilevel Measurement Error Autoregressive (MEAR) model, which separates true within-person fluctuations from occasion-specific fluctuations, including any random measurement error [24,25]. In the MEAR framework, each observation is decomposed into (1) a person-specific mean that is stable across time, (2) a systematic dynamic process over time reflecting true within-person variation, and (3) a measurement error term, assumed to be serially uncorrelated [25]. The dynamic process is modeled with a first-order autoregressive, AR(1), structure, in which each observation is regressed on the preceding observation [24,25]. The autoregressive effect depends on the time interval between successive observations, and the AR(1) process therefore assumes equal time intervals between all observations [26]. However, the data were irregularly spaced due to random sampling within time blocks, which may bias the estimate. Therefore, we added timestamped missing data to approximate regular hourly intervals at daytime, pausing the process overnight [26], following the procedure of Leertouwer et al. [27].

Within-person reliability is then defined as the proportion of the within-person variance attributable to the systematic autoregressive process [25]. The multilevel MEAR model allows these variance components to vary across persons, enabling estimation of person-specific reliabilities as well as an average within-person reliability [25]. We derived the within-person reliabilities from the models and computed the standard error of measurement (SEM). In addition, we derived the between-person reliability, defined as the proportion of total variance attributable to stable differences in person-specific means, which is equivalent to the traditional ICC [25].

#### 2.6.3. Measurement Reactivity

Repeated assessments can influence participants’ thoughts, emotions, and behavior, potentially altering the constructs being measured over time or the way these constructs are self-reported [28,29]. To evaluate measurement reactivity, we regressed momentary fatigue ratings on study day, modeled as a 7-level categorical variable, to test for systematic change across the 7-day EMA period. Any systematic change across days would indicate reactivity to the ongoing self-monitoring process.

#### 2.6.4. Construct Validity

Convergent validity was examined at the person level by testing whether higher baseline fatigue severity, as measured by the FSS, was positively associated with higher average momentary fatigue ratings across the study period. FSS was entered as a person-level predictor in a multilevel model fit to the total sample, including group (CP vs. control) as a covariate to account for group differences in fatigue. A standardized effect size was computed by multiplying the unstandardized coefficient by the ratio of the *SD* of FSS to the between-person *SD* of momentary fatigue. The latter was estimated by the random intercept from the unconditional three-level model, which captures the total variability between persons in their average momentary fatigue.

#### 2.6.5. Software

Multilevel models were performed using the lme4 package in R 4.5.0 [30], except for the MEAR models, which were run in JAGS using the rjags package for R, using the code provided by Leertouwer et al. [27]. Graphs were performed using the ggplot2 package.

### 2.7. Qualitative Analysis

Transcripts were analyzed thematically in relation to acceptability and perceived burden and reactivity to the EMA procedure, and relevance, clarity, and comprehensiveness of the items. Comments on the technical aspects of the app were outside the scope of the study and thus excluded in the analysis.

### 2.8. Patient and Public Involvement

Two adults with CP were consulted during study preparation and provided informal feedback on the relevance of the research topic and on the EMA survey, specifically. No individuals with CP were involved as investigators or co-authors.

## 3. Results

Ten participants with CP and eight controls were recruited for the study between December 2023 and September 2024. Twelve eligible adults with CP were informed about the study, and two of those declined participation (consent rate = 83%). The reasons for declining were reluctance to engage in frequent phone use (*n* = 1) and unspecified personal circumstances (*n* = 1). The consent rate in the control group was 100%. All participants in both groups completed the study (100% retention rate).

Sample characteristics are presented in Table 1. Control participants tended to be higher educated and more frequently in ordinary employment than participants with CP. All participants self-identified as being of Danish ethnicity. Everyone owned a smartphone, used it daily, and utilized their personal device for the study. Ten participants chose the standard schedule (07:30–22:00), seven chose the late (08:30–23:00), and one chose the early (06:30–21:00).

### 3.1. Feasibility

#### 3.1.1. Response Compliance

The average response rate in the CP group was 76% (527 responses out of 698 prompts), corresponding to a mean of 53 responses/person and 7.6 responses/day (the total prompt count excludes 2 scheduled assessments lost or delayed due to technical issues). The response rate ranged from 41% to 97% across participants with CP. Of the 527 initiated surveys, 510 (97%) were fully completed, yielding a full-completion rate of 73% (510/698). The response rate for the control group was 75%, ranging from 26% to 94%, and the full-completion rate was 73%.

**Figure 2:**
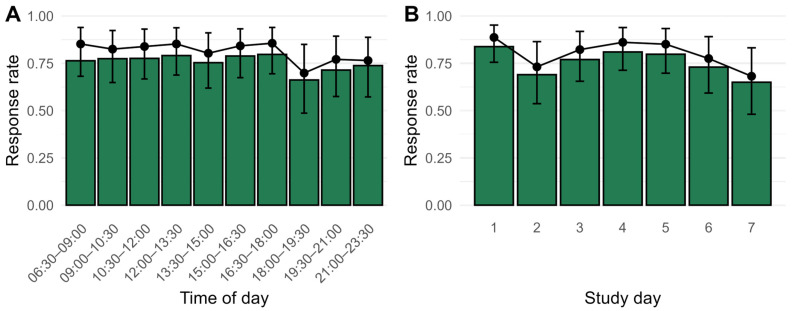
Compliance for the CP group relative to (**A**) time of day and (**B**) study day. Bars show observed average response rates, while points show the expected response rate for the average person on the average day with brackets indicating 95% confidence intervals.

For the CP group, the response rate did not vary significantly by time of day, Δ*χ*^2^(9) = 9.34, *p* = 0.41 (Figure 2, Panel A), but changed over study days, Δ*χ*^2^(6) = 16.15, *p* = 0.01, with drops in compliance on days 2, 6, and 7 (Figure 2, Panel B). Compliance did not differ between weekdays and weekends, Δ*χ*^2^(1) = 0.32, *p* = 0.57, and was also unrelated to fatigue at the previous assessment, Δ*χ*^2^(1) < 0.01, *p* = 0.98. Patterns were similar for the control group (test results are reported in Supplementary Materials: Tests on predictors of compliance in the control group).

#### 3.1.2. Observed Response Times

**Figure 3:**
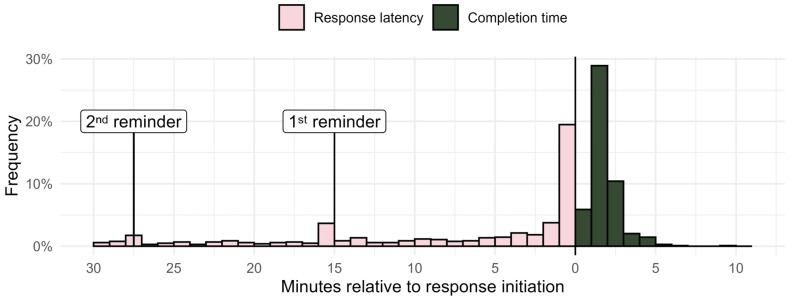
Observed response times in the CP group. Response latency, i.e., time from the first prompt to response initiation (x = 0) is shown in pink, and time spent completing surveys, i.e., from response initiation (x = 0) to submission, is shown in dark green.

In the CP group, the median response latency was 3 to 4 min. The mode was within the first minute after the prompt, and minor increases in response frequency were observed immediately after the first and second reminder (Figure 3). Longer response latency was unrelated to fatigue in that moment, Δ*χ*^2^(1) = 0.03, *p* = 0.86. The median time spent completing the survey was 98 s, ranging from 43 s to 9.6 min (Figure 3). The control group was slightly faster. The median response latency was between the second and the third minute after the prompt, and median completion time was 67 s (distributions are shown in Figure S1 in Supplementary Materials).

#### 3.1.3. Acceptability, Perceived Burden, Relevance, Clarity, and Comprehensiveness

Most participants with CP reported that the duration (7 days) and frequency (10 times/day) of assessments were acceptable. Some participants noted that 10 notifications per day were demanding and suggested either reducing the number of daily prompts, reducing the number of questions per prompt, or increasing the interval between prompts. Overall, the items were viewed as relevant and easy to answer, although some exceptions emerged. For example, the effort item was perceived as difficult to interpret, and response options for the motivation item (see Appendix A.2) were not mutually exclusive, as some activities could be seen as both obligatory and desired at the same time. A few item pairs were considered redundant by some participants, e.g., sad versus happy and energy versus fatigued, which some perceived as direct opposites. Four participants requested an open-ended comment field to elaborate on their responses, provide corrections, or note activities that were not recorded between assessments. Two participants also mentioned the lack of an item on irritability, which they felt was relevant to their experience of fatigue.

### 3.2. Intraindividual Variability

For the CP group, the mean score on momentary fatigue, averaged over days and persons, was 3.62 (*SE* = 0.60), with a between-person *SD* of 1.86 and a between-day *SD* of 0.64. For the fatigued item, 61% of the total variance was attributable to within-person variability and 57% was explained by moment-to-moment fluctuations within the day. Only 4% of the total variance reflected day-to-day variability within persons, indicating that daily averages were relatively stable. In contrast to fatigue, mood (i.e., sad, anxious, happy, and stressed) showed less intraindividual variability, with 17% of the variance in the sad item attributable to within-person variability. Meanwhile, contextual items (i.e., physical demand, mental demand, and sensory load) exhibited larger proportions of within-day variability, with 75% of the variance in physical demand occurring within the day. All model results are displayed in Table 2 for the CP group and Table S1 for the control group.

### 3.3. Reliabilities

In the CP group, the average within-person reliability of fatigued was 0.73, with individual estimates ranging from 0.66 to 0.90, indicating that 73% of the intraindividual variability was attributable to systematic changes over time rather than measurement error or occasion-specific fluctuations. The corresponding SEM averaged 1.13 points, 95% credible interval [0.68; 1.59], ranging from 0.68 to 1.54 across participants.

The estimated within-person reliability for fatigued was second highest among the set of EMA items in the CP group, exceeded only by sleepy (Table 3). Between-person reliability of fatigued within the CP group was 0.52. The within-person reliability of fatigued was lower in the control group compared to CP (Table 3).

### 3.4. Measurement Reactivity

Study day had no significant effect on momentary fatigue ratings in either the CP, Δ*χ*^2^(6) = 3.79, *p* = 0.71, or the control group, Δ*χ*^2^(6) = 5.34, *p* = 0.50, suggesting no evidence of reactivity to the self-monitoring process across study days (see Table S2). Additionally, participants with CP reported that self-monitoring did not affect their mood, daily routine, or social interactions, although the frequent sound notifications could be slightly disturbing or inappropriate in some social situations. One participant was a bit bothered by completing the same questions so many times a day, while another found joy in completing the survey.

### 3.5. Construct Validity

Momentary fatigue was positively associated with baseline FSS scores after controlling for group differences between CP and control, *b* = 0.56 (*SE* = 0.30), *β* = 0.51, Δ*χ*^2^(1) = 3.90, *p* = 0.048 (see Table S3). Thus, persons scoring one point higher on the FSS reported momentary fatigue levels that were on average 0.56 points higher across their repeated daily assessments, independent of having CP or not.

## 4. Discussion

The aim of this study was to evaluate feasibility and measurement properties of high-frequency EMA of fatigue in daily life of adults with CP (GMFCS I–III and CFCS I–II). Across several indicators, the findings provide preliminary support for the feasibility, reliability, and validity of this EMA protocol, while highlighting revisions that may reduce burden and increase compliance in a future large-scale study.

### 4.1. Feasibility

All participants completed the full study period with no dropouts, which is an important indicator of feasibility for this population. The average response rate was 76% for the CP group, broadly consistent with the reported average of 79% across 347 EMA studies in various populations [31] and 77% across 35 studies in acquired brain injury [32]. The only previous EMA study in adults with CP, to the authors’ knowledge, obtained a response rate of 78% using a single fatigue item delivered four times daily via SMS text message over seven days [19]. The present study achieved a comparable response rate, despite requiring participants to complete 20 items ten times daily, which is far more demanding. Taken together, these findings indicate that adults with CP can engage with high-frequency EMA effectively across the day. That said, the protocol was perceived as demanding by some participants, and thorough preparation and briefing of participants will be important for future studies. A slight decline in compliance toward the end of the monitoring period was observed, though this is a common pattern in EMA research [32] and did not compromise overall response rates or the representativeness of daily sampling.

The median survey completion time was 98 s for participants with CP, amounting to approximately 16 min per day across ten assessments. Furthermore, 97% of initiated surveys were fully completed, indicating that survey length was manageable even under the high sampling rate. For context, the 98-s completion time falls within the range of typical smartphone interactions, which rarely exceed two minutes [33], suggesting that individual assessments were consistent with habitual smartphone use patterns.

### 4.2. Intraindividual Variability and Reliability

More than half of the total variability in momentary fatigue was attributable to within-day fluctuations, indicating that fatigue is a highly dynamic symptom in adults with CP. Notably, the proportion of within-day variability exceeded that observed for emotional variables, suggesting that fatigue is more versatile than emotional states. Meanwhile, fatigue remained less immediately responsive than contextual variables, which is theoretically coherent, as the physical and mental demand and sensory load shift frequently as individuals move through their daily activities. These findings emphasize the value of intensive repeated measurement, as retrospective or single-timepoint assessments would fail to capture this intraindividual variability, which carries direct relevance for clinical management [3,6]. The mechanisms driving within-day fluctuations remain incompletely understood. Both physical and cognitive impairments and demands of daily life are likely to contribute in a complex interaction that may vary across individuals [6]. Future research mapping these factors would support translation into targeted fatigue management strategies. Both clinical practice and intervention trials could therefore benefit from incorporating EMA into outcome evaluation, enabling assessment of treatment effects on symptom dynamics.

Within-person reliability reflects the proportion of intraindividual variability that represents systematic, true fluctuations in the construct rather than measurement error. This property is critical for the meaningful interpretation of within-person change over time. The within-person reliability of momentary fatigue was estimated at 0.73 in the CP group, indicating that nearly three quarters of the variability reflect genuine changes in fatigue over time, and exceeding the estimate for most other items in this study. Directly comparable studies employing the MEAR framework are scarce, particularly for fatigue, but previously reported values have ranged from 0.50 to 0.70 for daily mood and 0.40 to 0.60 for positive affect [25]. Against these benchmarks, the present estimate is encouraging and provides preliminary support for the suitability of the item for measuring temporal fatigue dynamics in CP.

The SEM of 1.13 implies that the 95% confidence interval around an individual’s true score spans approximately ±2.21 points on the 0–10 scale, assuming normally distributed errors. Whether this precision is adequate depends on the magnitude of change considered meaningful, which remains to be established for momentary fatigue in this population. One available reference point is a study in multiple sclerosis that identified a minimal clinically important difference (MCID) of 0.94 to 1.04 points for momentary fatigue intensity on a 0–10 scale [34]. However, this estimate is not directly comparable, as it was derived from mean scores aggregated over seven-day periods before and after an intervention rather than from individual within-day ratings. Establishing a MCID for momentary fatigue in CP represents an important direction for future work to aid the interpretation of momentary fatigue ratings.

Given the small sample, the reliability and SEM estimate should be interpreted with caution, and replication in larger samples is warranted. Finally, the reliability estimate should be regarded as a conservative lower bound, since transient fluctuations in fatigue that are unique to a single measurement occasion are absorbed into the error term rather than the systematic dynamic process [25]. Consequently, the observed reliability likely underestimates the true reliability of the measure, depending on the timescale over which fatigue fluctuates [25], which is yet to be uncovered. It follows that reducing sampling frequency in future studies risks attenuating the reliability estimate further.

### 4.3. Measurement Reactivity and Validity

Measurement reactivity poses a threat to the validity of EMA studies, as it would undermine the assumption that changes over time reflect naturally occurring fluctuations in the construct. In this study, reactivity was examined both statistically and qualitatively. Neither approach yielded evidence of reactivity, as fatigue levels did not systematically shift across study days, and although some participants reported that sound notifications were occasionally inconvenient, none reported disruption to their everyday life or adaptation to the self-monitoring process. These findings cautiously support the interpretation that the data reflect genuine fluctuations in fatigue rather than procedural artefacts.

Turning to convergent validity, person-mean momentary fatigue was strongly associated with the FSS at baseline. This finding indicates that the simple question “how fatigued do you feel right now?” captures not only intraindividual variation in fatigue but also between-person differences in self-reported severity and functional impact of fatigue. A similar pattern has been reported in acquired brain injury, where aggregated momentary fatigue ratings were strongly associated (*r* = 0.63) with FSS scores obtained immediately following six days of EMA [35], suggesting that convergence between momentary and retrospective fatigue measures may generalize across neurological populations. Taken together, the absence of reactivity and the convergence with an established measure of fatigue severity offers preliminarily support for the overall validity of the EMA fatigue item.

Importantly, non-responses were unrelated to the level of fatigue reported at the preceding assessment, suggesting that missing data were not driven by fatigue, which otherwise could have biased the data.

### 4.4. Proposed Amendments for the Main Large-Scale Study

Initiatives for reducing the burden in future large-scale studies are important to maximize compliance and validity. For the main study, we propose adjusting the timing of assessments by increasing the intervals between successive assessments, while reducing the random sampling blocks from 60 to 30 min. That will yield more consistently spaced assessments at larger intervals, and participants will not receive a new prompt right after completing an assessment, which was a source of burden. Other strategies for reducing burden such as reducing the number of assessments per day is undesirable, since most of the variability in fatigue occurred within the day and that may reduce reliability and the potential for capturing short-term dynamics. A high sampling frequency is essential, so long as the timescale of fatigue fluctuations is unknown. Moreover, the relatively short seven-day period compensated to some extent for the high sampling frequency.

Although the items overall were clear and relevant, some revisions are warranted based on the interviews. First, the motivation item will be redesigned as a slider scale item, assessing the degree of motivation toward the current activity, instead of a multiple-choice item. Second, an optional open-ended comment field and an item on irritability will be incorporated, in response to participant feedback, and the stressed item will be dropped instead to avoid expanding the survey further. Finally, the relaxed item will be replaced by refreshed, as the latter more precisely targets the subjective experience of physical and mental restoration. Relaxed may conflate a behavioral state with a restorative feeling, since one may feel energized and refreshed without engaging in relaxing activities.

Finally, the main study will add concurrent measurements of sleep and physical activity using sleep diaries and actigraphy, and participants will be offered personalized feedback on their EMA data to incentivize compliance.

### 4.5. Limitations

Several limitations warrant consideration. First, the sample size is small, and estimates of model parameters should be regarded as preliminary and interpreted with caution until replicated in larger samples.

Second, the recruitment strategy may have introduced selection bias in both groups, thereby limiting the generalizability of the findings. For example, participants recruited through the researchers’ professional and social networks and individuals with CP who volunteer for research may be more committed to participation, leading to higher compliance than the broader target population.

Third, the sample is not representative of the whole CP population. Individuals with more severe impairments in gross motor function (GMFCS levels IV–V) or communication (CFCS levels II–V) were not included, and the EMA protocol in its current implementation in the SEMA^3^ app may not be feasible for these subgroups without modifications. Sensorimotor, visual, or cognitive impairments may hinder both compliance and participation, and adapting the protocol to be inclusive of the full severity spectrum of CP represents an important priority for future research. It should be noted, however, that the sample included individuals both with and without fatigue complaints, which supports generalizability across at least the higher-functioning segment of the CP population (i.e., GMFCS I–III and CFCS I–II).

Fourth, the survey was developed and validated in Danish, and due to the brevity of EMA items, where each word carries considerable semantic weight, even minor differences in meaning across languages may have implications for construct validity. For example, the English term “fatigue” has no direct Danish equivalent, and so the Danish term “træt” was used, which is the ordinary word for everyday tiredness and may therefore carry a less severe connotation than fatigue. The English version reported here was produced for reporting purposes only. Implementing the survey in another language would require a systematic forward-and-backward translation procedure and cross-cultural testing to ensure equivalence and support international comparability of findings.

Fifth, participants used their own smartphones for the study, which may have minimized disruption to daily routines, although variability in devices, operating systems, and notification settings could have influenced compliance and response times.

Finally, no individuals with CP were involved as research partners in the present study. To address this limitation, the planned main study will include an advisory panel consisting of people with CP to inform study design and interpretation.

## 5. Conclusions

High-frequency EMA of fatigue is feasible in adults with CP, and the intensive repeated assessments are justified by the substantial proportion of variability occurring within days. Explorative psychometric analyses suggest that the observed fluctuations are largely systematic rather than attributable to measurement error, and their convergence with baseline fatigue severity provides preliminary support for the validity of the item. Altogether, this pilot study provides initial proof-of-concept for the use of intensive EMA to capture the temporal dynamics of fatigue in adults with CP. The methodological amendments proposed based on these findings inform a future large-scale study, representing a first step toward understanding the mechanisms underlying daily fatigue fluctuations and ultimately advancing fatigue management in this population.

## Figures and Tables

**Table 1:** Demographic and clinical characteristics of the total sample and by group.

Variable	Total Sample (*N* = 18)	By Group		
		Cerebral Palsy (*n* = 10)	Control (*n* = 8)	*p*
Gender, female, *n* (%)	11 (61%)	6 (60%)	5 (62%)	1.00
Age, *M* (*SD*)	41.4 (10.6)	43.7 (11.8)	38.6 (8.7)	0.35
Level of education, *n* (%) ^a^				0.05
Elementary education	2 (12%)	1 (10%)	1 (14%)	
Upper secondary education	5 (29%)	5 (50%)	0 (0%)	
Higher education	10 (59%)	4 (40%)	6 (86%)	
Occupation, *n* (%) ^a^				0.06
Ordinary employment	11 (65%)	4 (40%)	7 (100%)	
Supported employment	2 (12%)	2 (20%)	0 (0%)	
Unemployed	2 (12%)	2 (20%)	0 (0%)	
Retired	2 (12%)	2 (20%)	0 (0%)	
Civil status, *n* (%) ^a^				0.62
Married/partner	10 (59%)	5 (50%)	5 (71%)	
Single	7 (41%)	5 (50%)	2 (29%)	
Living arrangement, *n* (%) ^a^				0.42
Living alone	5 (29%)	4 (40%)	1 (14%)	
Living with others	11 (65%)	5 (50%)	6 (86%)	
Housing facility	1 (6%)	1 (10%)	0 (0%)	
FSS, mean (*SD*) ^a^	3.5 (1.5)	4.1 (1.6)	2.7 (0.9)	0.07
GMFCS, *n* (%)				
Level I	-	6 (60%)	-	
Level II	-	2 (20%)	-	
Level III	-	2 (20%)	-	
CFCS, *n* (%)				
Level I	-	10 (100%)	-	

Note. FSS = Fatigue Severity Scale; GMFCS = Gross Motor Function Classification System; CFCS = Communication Function Classification System. The Mann–Whitney U test was used to compare continuous variables and Fisher’s exact test for categorical variables. ^a^ One missing value in the control group.

**Table 2:** Estimates for empty 3-level models on EMA items in the CP group.

Item	*N* (Level 1)	Fixed Effect	Random Effects, *SD*			Intraclass Correlation Coefficient (ICC)		Model Fit
		Intercept, Est. (*SE*)	Person Intercepts	Person: Day Intercepts	Residual	Person (Level 3)	Person: Day (Level 2)	AIC
Fatigued	524	3.62 (0.60)	1.86	0.64	2.29	0.38	0.04	2424.31
Energy	525	6.47 (0.58)	1.81	0.50	1.88	0.46	0.04	2223.99
Exhausted	524	3.02 (0.75)	2.32	0.84	2.01	0.53	0.07	2317.94
Effort	520	4.42 (0.71)	2.23	0.53	1.97	0.54	0.03	2253.66
Sleepy	524	3.19 (0.64)	1.95	0.82	2.36	0.38	0.07	2467.17
Pain	523	1.31 (0.45)	1.38	0.63	1.02	0.57	0.12	1633.27
Concentration	523	6.42 (0.48)	1.49	0.38	1.68	0.43	0.03	2092.04
Happy	522	7.50 (0.71)	2.20	0.87	1.07	0.72	0.11	1717.71
Relaxed	523	6.38 (0.56)	1.74	0.54	1.95	0.43	0.04	2250.28
Sad	522	1.77 (0.71)	2.24	0.69	0.79	0.82	0.08	1413.12
Stressed	521	2.37 (0.68)	2.12	0.36	1.44	0.67	0.02	1935.16
Anxious	521	2.39 (0.80)	2.52	0.62	1.11	0.80	0.05	1715.28
Physical demand	513	2.70 (0.46)	1.40	0.11	2.46	0.24	0.00	2417.00
Mental demand	512	3.93 (0.61)	1.90	0.40	2.28	0.41	0.02	2348.61
Sensory load ^a^	511	1.36 (0.21)	0.65	0.14	0.90	0.33	0.02	1397.76

Note. Method = Restricted Maximum Likelihood estimation. Persons (Level 3) = 10; Days within persons (Level 2) = 70. Random effects are reported in standard deviations. Est. = Estimate; *SE* = Standard error; AIC = Akaike Information Criterion. ^a^ The scale ranged from 0 to 4, while the other scales ranged from 0 to 10.

**Table 3:** Reliabilities derived from multilevel measurement error autoregressive (MEAR) models.

Item	CP (*n* = 9) ^a^			Control (*n* = 8)		
	${rel}_{w}$	${SEM}_{w}$	${rel}_{b}$	${rel}_{w}$	${SEM}_{w}$	${rel}_{b}$
Fatigued	0.73	1.13	0.52	0.50	1.12	0.59
Energy	0.64	1.10	0.61	0.56	0.81	0.36
Exhausted	0.61	1.14	0.63	0.55	0.90	0.74
Effort	0.41	1.51	0.65	0.36	1.33	0.52
Sleepy	0.74	1.17	0.51	0.57	1.11	0.56
Pain ^b^	0.59	0.71	0.66	0.56	0.51	0.61
Concentration	0.53	1.13	0.60	0.24	1.17	0.49
Happy	0.47	0.80	0.75	0.55	0.74	0.44
Relaxed	0.47	1.35	0.59	0.50	1.02	0.60
Sad ^c^	0.63	0.68	0.79	0.58	0.45	0.26
Stressed	0.45	1.02	0.77	0.47	0.74	0.49
Anxious	0.41	0.89	0.88	0.29	0.85	0.70
Physical demand	0.59	1.34	0.29	0.43	1.44	0.31
Mental demand	0.63	1.31	0.54	0.50	1.51	0.33

Note. ${rel}_{w}$ = the average person-specific reliability. ${SEM}_{w}$ = the average standard error of measurement for within-person fluctuations. ${rel}_{b}$ = between-person reliability. ^a^ Model failed to converge for one participant across all items. ^b^ Model converged for a total of seven participants in the control group. ^c^ Model converged for a total of seven participants in the CP group.

## Data Availability

The datasets presented in this article are not readily available because they contain non-anonymized human participant data and cannot be shared publicly due to ethical and legal restrictions. Requests to access the datasets should be directed to the corresponding author and are subject to ethical and institutional approval.

## Supplementary Materials

The following supporting information can be downloaded at: https://www.mdpi.com/article/10.3390/brainsci16050515/s1, Figure S1: Response time distributions in the control group; Table S1: Estimates for empty 3-level models on EMA items in the control group; Table S2: Models for measurement reactivity analyses; Table S3: Models for construct validity analyses.

## Abbreviations

The following abbreviations are used in this manuscript:

CP	Cerebral palsy
EMA	Ecological Momentary Assessment
MEAR	Measurement Error Autoregressive Model
GMFCS	Gross Motor Function Classification System
CFCS	Communication Function Classification System
FSS	Fatigue Severity Scale
ICC	Intraclass correlation coefficient
SEM	Standard error of measurement
SD	Standard deviation
SE	Standard error
AIC	Akaike Information Criterion
MCID	Minimal clinical important difference

## Appendix A

### Appendix A.1. EMA Items

Items are listed in Table A1 in the order in which they were administered. Questions addressing more transient experiences were administered first, followed by items addressing more stable and factual information. To minimize routinized or careless responding and to maintain attention, items 4 to 13 were randomized within their item set, whereas the remaining items were presented in a fixed order. Item 3 was administered only if item 2 was rated above 0 (using branching logic). Item 1 was assessed using a pictorial slider scale representing low (empty battery) or high (full battery) levels of energy [36]. Keywords were emphasized using bold and italics, as shown in Table A1, to facilitate quick comprehension and responding. The selected response option was displayed on the screen prior to submission. Participants were unable to change their response once an item had been submitted.

All items addressing fatigue and related factors, except items 3 and 6, were adapted from the Daytime Insomnia Symptom Scale [37]. The phrase “right now” was added to the questions to emphasize the momentary nature of the assessment, and response anchors were modified. Contextual items were adapted from the work of Csikszentmihalyi [38], the NASA Task Load Index [39], and EMA studies in acquired brain injury [40,41].

The English wording provided in Table A1 is a translation from the Danish version for reporting purposes. The original Danish prototype questionnaire is available from the corresponding author upon request.

**Table A1:** Item formulation and response options for EMA items.

No.	Item	Response Scale	Anchors (0; 10)	Ref.
1	How much ***energy*** do you have right now?	Slider (0–10)	Pictorial (empty/full battery)	[36]
2	How ***fatigued*** do you feel right now?	Slider (0–10)	No fatigue; Severe fatigue	[18,19,37]
3	How does fatigue ***feel*** right now?	Multiple choice	N/A	
4	How ***exhausted*** do you feel right now?	Slider (0–10)	Not at all; Very much	[37]
5	How much of an ***effort*** is it to do anything right now?	Slider (0–10)	None; Very much	[37]
6	How much ***pain*** do you have right now?	Slider (0–10)	No pain; Worst pain imaginable	NRS
7	How ***sleepy*** do you feel right now?	Slider (0–10)	Not at all; Very much	[37]
8	How well are you able to ***concentrate*** right now?	Slider (0–10)	Not at all; Very much	[37]
9	How ***happy*** do you feel right now?	Slider (0–10)	Not at all; Very much	[37]
10	How ***relaxed*** do you feel right now?	Slider (0–10)	Not at all; Very much	[37]
11	How ***sad*** do you feel right now?	Slider (0–10)	Not at all; Very much	[37]
12	How ***stressed*** do you feel right now?	Slider (0–10)	Not at all; Very much	[37]
13	How ***anxious*** do you feel right now?	Slider (0–10)	Not at all; Very much	[37]
14	What were you doing when you got the notification?	Multiple choice	N/A	[38]
15	Why were you doing this particular activity?	Multiple choice	N/A	[38]
16	How ***physically*** demanding is what you were doing?	Slider (0–10)	Not at all; Very much	[39]
17	How ***mentally*** demanding is what you were doing?	Slider (0–10)	Not at all; Very much	[39]
18	Where were you when you got the notification?	Multiple choice	N/A	[41]
19	How many ***sensory impressions*** were around you when you got the notification?	Likert-type	N/A	
20	Who were with you when you got the notification?	Multiple choice	N/A	[40]

Note. Response options for multiple choice questions are provided in the text below. Ref. = reference to the study, from which the item was adapted. NRS = Numerical Rating Scale of Pain.

### Appendix A.2. Response Options for Multiple Choice and Likert-Type Questions

Item 3: (a) Primarily physical; (b) Primarily mental; (c) Both; (d) Neither; (e) Not relevant.

Item 14: (a) Nothing (e.g., daydreaming, waiting); (b) Relaxing (e.g., resting, napping, sleeping); (c) Sedentary leisure activity (e.g., reading, phone, tv); (d) Active leisure activity (e.g., workout, walk, sport); (e) Work tasks (work or study); (f) Self-care (e.g., showering, dressing, meals); (g) Household chores (e.g., cleaning, shopping, cooking); (h) Transport (e.g., driving, biking, train); (i) Socializing (e.g., talking, shared activity); (j) Other/Don’t know.

Item 15: (a) I had to; (b) I wanted to do it; (c) I had nothing else to do; (d) Not relevant.

Item 18: (a) At home; (b) At work or study; (c) At family’s or friend’s place; (d) Transport; (e) Public space; (f) Other.

Item 19: (1) Very low degree; (2) Low degree; (3) Some degree; (4) High degree; (5) Very high degree.

Item 20: (a) No one (alone); (b) spouse or partner; (c) family members; (d) friends; (e) pets; (f) co-workers; (g) other known people; (h) health care providers; (i) unknown people; and (j) people you are socializing with electronically.
