# Supplementary material for: Ecological Momentary Assessment of Fatigue in Adults with Cerebral Palsy: Feasibility, Reliability, and Validity
Source: Brain Sci. 2026 May 12;16(5):515. doi: 10.3390/brainsci16050515 (PMC13204188; doi:10.3390/brainsci16050515)
Supplement: Supplementary file 1 [file brainsci-16-00515-s001.zip › brainsci-4262593-supplementary.pdf]

Supplementary Materials

Ecological Momentary Assessment of Fatigue in Adults with Cerebral Palsy: Feasibility, Validity, and Reliability

Table of Contents

Tests on predictors of compliance in the control group 1
Figure S1: Response time distributions in the control group 1
Table S1: Estimates for empty 3-level models on EMA items in the control group 2
Table S2: Models for measurement reactivity analyses 2
Table S3: Models for construct validity analyses 3

Tests on predictors of compliance in the control group

In the control group, the response rate did not vary significantly by time of day, $\Delta\chi^2(9) = 8.27$, $p = .51$, study day, $\Delta\chi^2(6) = 10.93$, $p = .09$, weekday vs. weekend, $\Delta\chi^2(1) = 0.03$, $p = .87$, or lagged fatigue rating, $\Delta\chi^2(1) = 1.83$, $p = .98$.

Figure S1: Response time distributions in the control group

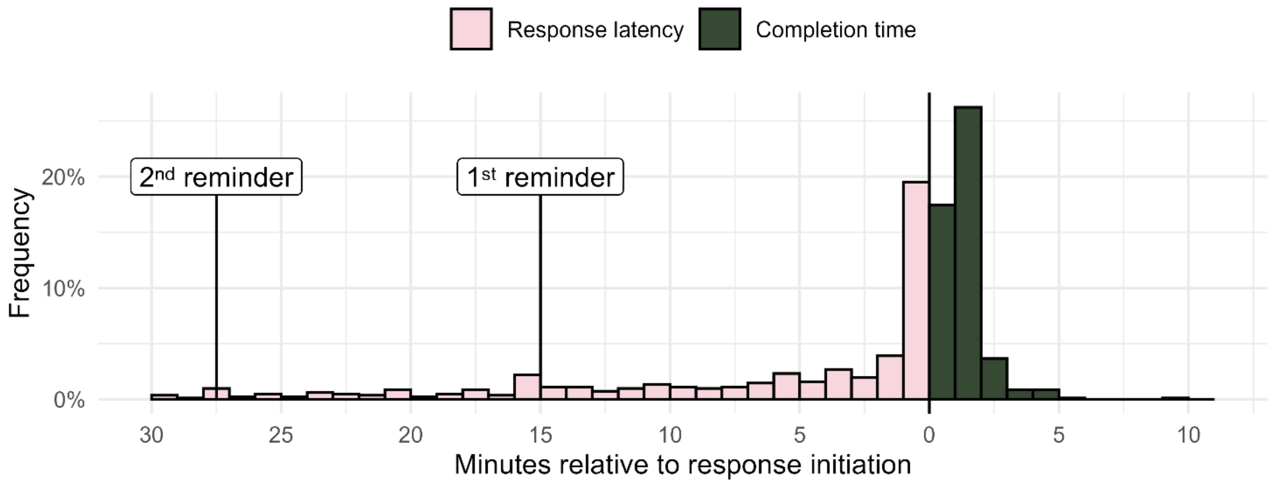

Note. Observed response times in the control group. Time from the first notification (x = 0) to response initiation is shown in pink, and the distribution of time spent completing surveys is shown in dark green.

Table S1: Estimates for empty 3-level models on EMA items in the control group

Item	N (Level 1)	Fixed effect	Random effects, *SD*			Intraclass Correlation Coefficient (ICC)		Model fit
		Intercept, Est. (*SE*)	Person intercepts	Person:Day intercepts	Residual	Person (Level 3)	Person:Day (Level 2)	AIC
Fatigued	412	2.62 (0.49)	1.33	0.61	1.52	0.4	0.08	1586.84
Energy	413	6.7 (0.25)	0.65	0.45	1.21	0.2	0.1	1389.87
Exhausted	409	2.48 (0.64)	1.79	0.65	1.35	0.59	0.08	1492.08
Effort	410	3.41 (0.46)	1.26	0.65	1.64	0.34	0.09	1640.02
Sleepy	411	2.45 (0.52)	1.42	0.56	1.61	0.41	0.06	1622.03
Pain	410	0.64 (0.26)	0.71	0.53	0.63	0.42	0.24	915.43
Concentration	412	6.46 (0.31)	0.84	0.5	1.23	0.29	0.1	1407.14
Happy	409	6.84 (0.28)	0.75	0.48	1.02	0.31	0.13	1256.38
Relaxed	411	6.48 (0.46)	1.27	0.51	1.37	0.43	0.07	1497.27
Sad	410	0.78 (0.48)	1.35	0.27	0.81	0.71	0.03	1065.32
Stressed	410	1.14 (0.32)	0.86	0.52	1.14	0.32	0.12	1350.39
Anxious	409	1.33 (0.48)	1.34	0.42	1.06	0.58	0.06	1284.3
Physical demand	404	1.73 (0.34)	0.91	0.59	1.84	0.18	0.08	1695.24
Mental demand	404	2.95 (0.41)	1.08	0.72	2.06	0.19	0.09	1790.65
Sensory load [a]	404	1.4 (0.17)	0.46	0.21	0.76	0.26	0.05	977.14

Note. Method = Restricted Maximum Likelihood estimation. Persons (Level 3) = 8; Days within persons (Level 2) = 56. Random effects are reported in standard deviations. Est. = Estimate; SE = Standard error; AIC = Akaike Information Criterion.

[a] The scale ranged from 0 to 4, while the other scales ranged from 0 to 10.

Table S2: Models for measurement reactivity analyses

	CP group		Control group	
**Fixed effects**	**Model 1**	**Model 2**	**Model 1**	**Model 2**
γ_{000} = Intercept, est. (SE)	3.6219 (0.5712)	3.84356 (0.63743)	2.619 (0.454)	2.20141 (0.52079)
γ_{010} = Day 2, est. (SE)		0.11211 (0.46158)		0.64200 (0.40045)
γ_{020} = Day 3, est. (SE)		-0.09681 (0.45189)		0.54082 (0.39383)
γ_{030} = Day 4, est. (SE)		-0.49280 (0.44652)		0.07724 (0.39618)
γ_{040} = Day 5, est. (SE)		-0.56575 (0.44953)		0.60237 (0.39112)
γ_{050} = Day 6, est. (SE)		-0.09398 (0.45690)		0.63726 (0.39798)
γ_{060} = Day 7, est. (SE)		-0.39591 (0.46625)		0.45432 (0.40043)
**Random part**				
Level-three variance: φ_0^2 = var(V_{00k})	3.0949	3.0761	1.5420	1.5549
Level-two variance: τ_0^2 = var(U_{0jk})	0.4049	0.3433	0.3731	0.3067
Level-one variance: σ_0^2 = var(R_{ijk})	5.2482	5.2444	2.3102	2.3063
AIC	2425.1	2433.3	1587.2	1593.8

ML estimation. For the CP group: Level 3 (persons) = 10; Level 2 (days within persons) = 70; Level 1 (observations) = 524. For the control group: Level 3 (persons) = 8; Level 2 (days within persons) = 56; Level 1 (observations) = 412.

Table S3: Models for construct validity analyses

Fixed effects	Model 1	Model 2	Model 3
γ_{000} = Intercept, est. (SE)	3.166 (0.4160)	2.5140 (0.6137)	2.9820 (0.5926)
γ_{001} = Group, est. (SE)		1.1089 (0.8009)	0.3102 (0.8117)
γ_{002} = FSS, est. (SE)			0.5625 (0.2695)
**Random part**			
Level-three variance: φ_0^2 = var(V_{00k})	2.8138	2.5142	1.9841
Level-two variance: τ_0^2 = var(U_{0jk})	0.3509	0.3513	0.3516
Level-one variance: σ_0^2 = var(R_{ijk})	3.9642	3.9640	3.9634
AIC	3996.4	3996.6	3994.7

Note. Method = Maximum Likelihood estimation. Level 3 (persons) = 17; Level 2 (days within persons) = 119; Level 1 (observations) = 919. To facilitate interpretation of parameters, FSS was mean-centered with a standard deviation of 1.53.
